# The Application of Virtual Reality in Shoulder Surgery Rehabilitation

**DOI:** 10.7759/cureus.58280

**Published:** 2024-04-15

**Authors:** Jihun Nam, Yong-Gon Koh, Sunghoon Chung, Paul S Kim, Jihoon Jang, Joon-Hee Park, Kyoung-Tak Kang

**Affiliations:** 1 Mechanical Engineering, Yonsei University, Seoul, KOR; 2 Orthopaedic Surgery, Yonsei Sarang Hospital, Seoul, KOR; 3 Orthopaedic Surgery, The Bone Hospital, Seoul, KOR; 4 Orthopaedics, Yonsei Siwon Orthopaedic Clinic, Seoul, KOR; 5 Anesthesiology & Pain Medicine, Hallym University College of Medicine and Kangdong Sacred Heart Hospital, Seoul, KOR; 6 Skyve R&D Lab, Skyve Co. LTD., Seoul, KOR

**Keywords:** mixed reality, oculus quest 2, rehabilitation, shoulder, virtual reality

## Abstract

To achieve a positive functional prognosis in orthopedic surgery, particularly in shoulder surgeries, effective rehabilitation is essential. Recently, there has been growing interest in the use of virtual reality (VR) in the field of orthopedics, particularly for preoperative education and training, as well as clinical and home-based rehabilitation. This report describes the process of developing an application utilizing Meta Quest 2 VR technology (Meta, CA, USA) for rehabilitation after shoulder surgery. This application assists patients in performing postoperative exercises at home by wearing VR equipment tailored to their postoperative weeks. The advantages of VR rehabilitation lie in overcoming the limitations of traditional rehabilitation methods and providing patients with a better rehabilitation experience. Moreover, automating the rehabilitation process and reducing patients' visits to clinics can lead to cost savings. This report raises expectations for the potential and scalability of VR utilization, extending beyond orthopedics to other fields. In addition, it anticipates that with better feedback and motivation, the rehabilitation effects for patients can be further enhanced.

## Introduction

Traditionally, shoulder function has been assessed using clinical measures such as strength, pain, and range of motion (ROM) [1]. Postsurgery, the postoperative pain and discomfort can significantly impact the patient's daily activities and overall quality of life. Rehabilitation plays a key role in maximizing recovery and improving function to enhance performance and autonomy in daily activities life after shoulder surgery [2]. Patients who undergo shoulder joint surgery typically need to make periodic visits to the clinic or perform exercises at home tailored to upper limb movements, ranging from 2 to 48 weeks. However, there are limitations to performing rehabilitation exercises at home using only pictures or videos. The most significant issue is the inability to confirm whether the patient is executing the correct rehabilitation movements.

Recently, there has been growing interest in the use of virtual reality (VR) for rehabilitation after orthopedic surgery in both clinical and home settings [3]. Among these advantages are enhanced patient motivation, accelerated training effects, and the potential to adjust movement strategies through the feedback features of activities [4]. Specifically, the most significant advantage of using VR in rehabilitation is that it can provide immediate feedback on whether individuals are executing the correct movements.

VR involves simulating experiences through physical hand devices and computer-generated software combination, creating immersive and interactive environments to simulate reality [5]. It is currently a commonly accessible device that can be easily used at home [6,7]. The immersiveness of these devices enables users to experience various virtual environments, facilitating interactive patient experiences. This can make long-term rehabilitation more enjoyable and engaging [8]. Lohre et al. conducted a review study on VR in shoulder and elbow surgery, emphasizing the importance of focusing on the development, validation, and implementation of high-quality immersive VR products [9]. Recently, Carnevale et al. evaluated the accuracy of Meta Quest 2 (Meta, CA, USA) VR devices from the perspective of shoulder rehabilitation and determined that they offer a level of accuracy acceptable for rehabilitation exercises [10].

This report outlines the process of developing a shoulder rehabilitation application using Meta Quest 2.

## Technical report

Shoulder surgery classification and selection of rehabilitation exercises

The scope of rehabilitation therapy was defined within the context of shoulder surgery. Initially, shoulder surgeries were categorized into debridement, labral repair, rotator cuff repair, and arthroplasty. Rotator cuff repair was further subdivided into small-medium tears and large-massive tears, resulting in a total of five surgical types. Subsequently, rehabilitation exercises were delineated. They were broadly divided into three categories: postoperative exercises, ROM exercises, and strength exercises. Postoperative exercises comprised eight movements (how to wear assistive device, shoulder shrugs exercise, shoulder forward rotation exercise, shoulder backward rotation exercise, hand stretching exercise, wrist stretching exercise, elbow stretching exercise, hold the wrist and rotate the shoulders outward exercise), ROM exercises consisted of 14 movements (arm lifting exercise, shoulder stretch, internal rotation exercise, outward arm rotation stretch, T-bar assisted arm stretch behind the back, T-bar assisted arm outward rotation stretch, desk based shoulder exercises, external rotation exercise, seated horizontal table slide, seated shoulder elevation with cane, sleeper stretch, sidelying horizontal adduction stretch, active assistive shoulder flexion, shoulder flexion with cane), and strength exercises encompassed 17 movements (Standing abduction, proprioceptive neuromuscular facilitation (PNF) D1 diagonal lifts, PNF - D2 diagonal lifts, field goals, scap retraction, sidelying external rotation, sidelying abduction to 90 degrees, external rotation at 90 deg abduction, flexor strengthening exercise, shoulder extension strengthening exercise, rows with resistance band, adductor strengthening exercise, external rotator strengthening exercise, abduction muscle strengthening exercise, W exercise with resistance band, resistance band dynamic hug, resistance band bicep curls). All rehabilitation exercises for each surgery were uniformly structured, albeit with varying durations of rehabilitation periods (Table 1) [11-13].

**Table 1:** Rehabilitation exercise for each surgery ROM: Range of motion; RCT: rotator cuff tear

	Debridement	Labral repair	RCT		Arthroplasty
			Small-medium	Large-massive	
Postoperative exercise	2 weeks	4 weeks	4 weeks	6 weeks	6 weeks
ROM exercise	2 weeks	4 weeks	4 weeks	6 weeks	6 weeks
Strengthening exercise	4 weeks	12 weeks	12 weeks	16 weeks	8 weeks

Avatar creation and animation implementation

**Figure 1:**
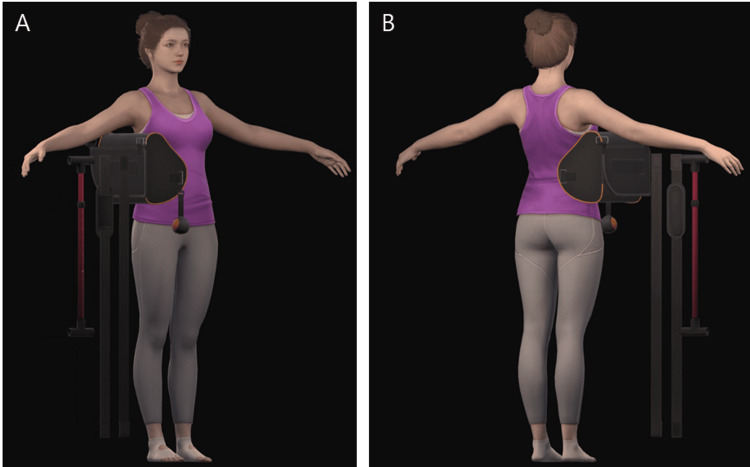
The avatar performing rehabilitation movements. (A) Front view and (B) rear view.

We have developed an avatar to demonstrate rehabilitation movements (Figure 1). When the patient wears VR glasses, the avatar is displayed in front of their eyes. Since the avatar is created in 3D, the patient can observe its movements by maneuvering around it. The avatar was produced using 3ds Max (Autodesk, CA, USA).

**Figure 2:**
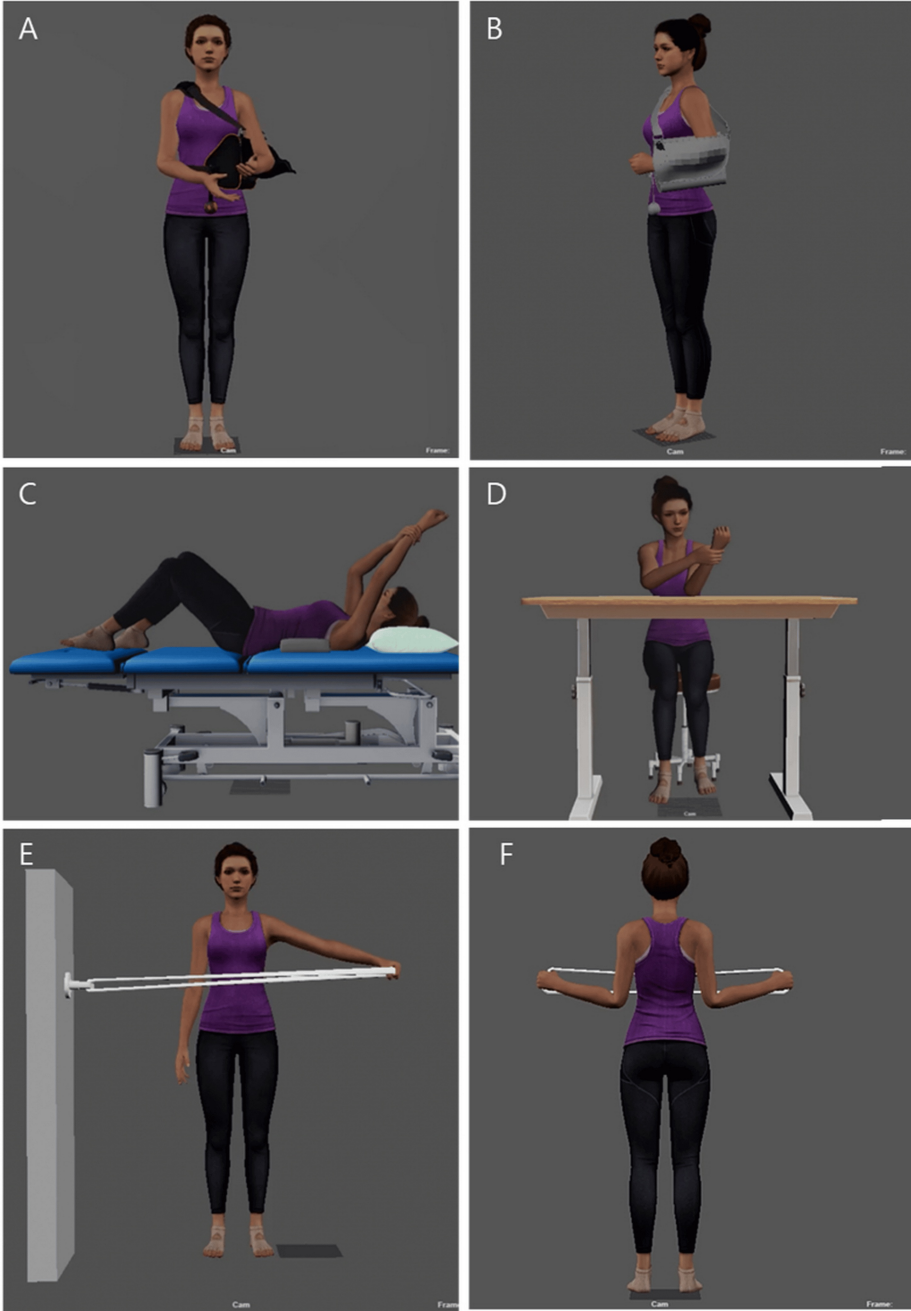
Captured screenshots of rehabilitation exercises: Postoperative exercises (A, B), range of motion (ROM) exercises (C, D), and strengthening exercises (E, F).

Animations depicting rehabilitation movements were created using the completed avatar. The rehabilitation exercises consist of eight postoperative exercises, 14 ROM exercises, and 17 strength exercises. These animations were crafted by animation implementation specialists, incorporating feedback from both shoulder surgery specialists and rehabilitation experts into each movement. Figure 2 displays captured screens of the rehabilitation exercises. Additionally, tools such as beds and desks necessary for performing the rehabilitation movements were generated and utilized.

Avatar VR visualization and GUI implementation

The avatar created using the Unity video game engine (Unity Technologies, CA, USA) was visualized on the VR screen. The VR equipment utilized was the Meta Quest 2.

After shoulder surgery, the rehabilitation protocol program used is Sagarvision VR (Skyve, Seoul, Korea). An application was developed to enable patients to perform rehabilitation exercises based on the type of surgery received and the current postoperative week. The application is structured into three stages. Firstly, the type of surgery is selected from options including debridement, labral repair, rotator cuff repair (small-medium), rotator cuff repair (large-massive), and arthroplasty. After selecting the surgery, the postoperative week is chosen. Upon selecting the week, the rehabilitation exercises for that specific week, as outlined in Table 1, commence. As the avatar initiates the movements, the patient follows along. The GUI screen provides buttons for returning to the initial screen, pausing, replaying, advancing to the next movement, and exiting.

## Discussion

Summary

In this study, we developed an application that applies VR to shoulder rehabilitation. This application provides rehabilitation programs for five types of surgery: debridement, labral repair, rotator cuff repair (small-medium), rotator cuff repair (large-massive), and arthroplasty. Patients can wear VR glasses and choose appropriate exercises for the postoperative week to proceed with their rehabilitation.

The advantages of rehabilitation using VR

Rehabilitation is a long-term process involving interventions such as physical therapy or psychological therapy [14]. Patients who have undergone shoulder surgery are mandated to attend regular clinic visits for rehabilitation exercises, spanning from two weeks to a maximum of 48 weeks, or engage in home-based rehabilitation. This rehabilitation approach imposes burdens on patients in terms of time and cost. Moreover, using images or videos to follow rehabilitation exercises at home has limitations in accurately identifying the rehabilitation movements. Some promising results have been demonstrated by rehabilitation methods based on VR [15]. Previous research has predominantly focused on investigating the effectiveness of VR exergaming in neurological disorders [4,16-18] or among elderly populations [19-22], with an emphasis on balance-related outcomes.

The VR rehabilitation application developed in this study allows patients to mimic movements by observing the three-dimensional movements of the avatar. Patients can learn rehabilitation exercises by moving their bodies and viewing desired perspectives. Utilizing such VR systems enables the automation of the rehabilitation process and can be performed without the supervision of rehabilitation therapists, thereby reducing cost burdens. Additionally, patients can perform rehabilitation at home, thus reducing the burden of clinical visits [14].

Limitations and future directions

Extend to Mixed Reality (MR)

The drawback of VR rehabilitation is that elderly patients may find it difficult to operate rehabilitation equipment. Furthermore, without visibility of their surroundings, there is a risk of collision with nearby objects if sufficient space is not secured. Therefore, for safety reasons, patients' rehabilitation activities are feasible to be conducted at home, with a preference for them to be performed in the presence of a caregiver or under supervision. If patients can see not only avatars but also their own bodies when performing rehabilitation movements, more accurate feedback on the movements is possible. Recently, Meta announced the Meta Quest 3 or Vision Pro (Apple, CA, USA). This device is equipped with MR functionality. With this feature, patients can perform rehabilitation movements while viewing their own bodies and the actual surrounding environment. The application developed in this study can be extended to MR, enabling patients to perform rehabilitation movements while observing their own bodies.

Addition of Angle Measurement Functionality Using Inertial Measurement Unit (IMU) Sensors

The greatest advantage of rehabilitation using VR is the ability to assess angles using the physical hand device provided by the equipment. This allows for immediate feedback through VR, informing whether the patient is performing the correct angle movements. Therefore, achieving successful rehabilitation outcomes becomes more feasible if the angles of the patient's joints can be measured and provided with feedback during these movements. After shoulder surgery, some movements may not allow the use of a physical hand device due to the utilization of specific assistive devices. This is because it prevents the measurement of the patient's joint ROM, making immediate feedback impossible. Installing IMU sensors on the wrist, forearm, and other areas allows for accurate angle measurements without the need for a physical hand device. In the subsequent development stages, we plan to incorporate joint angle calculation functionality using these sensors. This will enable patients to receive feedback on their current angles and be motivated to achieve the desired angles.

Motivation Through Gamification

In particular, in the field of orthopedic surgery, rehabilitation following shoulder surgery is not merely exercise but requires intricate treatment. Because postshoulder surgery rehabilitation involves daily, consistent efforts to gradually expand the ROM, emphasizing isokinetic motion, and focusing on functional recovery. Therefore, motivating patients can often be challenging. Motivation through gamification is a crucial element that can make the rehabilitation process more engaging and rewarding. In the course of history, gaming companies have offered traditional video game hardware at very affordable prices. However, they later generate significant profits from software sales. Following the same pattern as traditional video game marketing, major technology companies are mass-producing millions of VR devices each year at low costs. Therefore, while VR devices continue to become more affordable, the quality is rapidly advancing. For these reasons, the most crucial element in rehabilitation programs utilizing VR can be identified as motivation through the gamification of software. By incorporating various gaming elements, patients can be motivated to perform rehabilitation exercises more enjoyable and consistently participate. Elements such as challenge tasks and reward systems, level and experience point systems, community and competition, storytelling, assessment, and feedback can be effectively utilized to enable patients to engage in rehabilitation activities like games and sustain continuous participation.

## Conclusions

This paper introduces the development of a VR application for shoulder rehabilitation using the Meta Quest 2 device. This application provides a rehabilitation program for patients after shoulder surgery, allowing them to perform tailored exercises using VR headsets. The advantages of VR rehabilitation lie in overcoming the limitations of traditional rehabilitation methods and providing patients with a better rehabilitation experience. Additionally, automating the rehabilitation process and reducing patients' clinic visits can lead to cost savings. Therefore, the utilization of VR in rehabilitation shows great promise for further advancement and scalability. This is seen as a promising avenue to enhance individual adherence to long-term rehabilitation programs by providing more accurate feedback and motivation. In future research endeavors, it is crucial to devise precise and objective methodologies for evaluating the clinical effectiveness of emerging technologies. Additionally, the benefits of VR in telerehabilitation should be distinctly demonstrated compared to conventional face-to-face orthopedic rehabilitation.
