# Correction: The Application of Virtual Reality in Shoulder Surgery Rehabilitation 

**DOI:** 10.7759/cureus.c296

**Published:** 2025-09-16

**Authors:** Jihun Nam, Yong-Gon Koh, Sunghoon Chung, Paul S Kim, Jihoon Jang, Joon-Hee Park, Kyoung-Tak Kang

**Affiliations:** 1 Mechanical Engineering, Yonsei University, Seoul, KOR; 2 Orthopaedic Surgery, Yonsei Sarang Hospital, Seoul, KOR; 3 Orthopaedic Surgery, The Bone Hospital, Seoul, KOR; 4 Orthopaedics, Yonsei Siwon Orthopaedic Clinic, Seoul, KOR; 5 Anesthesiology & Pain Medicine, Hallym University College of Medicine and Kangdong Sacred Heart Hospital, Seoul, KOR; 6 Skyve R&D Lab, Skyve Co. LTD., Seoul, KOR

The Ethics Statement and Conflict of Interest Disclosures section has been updated to disclose the following:

**Financial relationships:** Kyoung-Tak Kang declare(s) employment from Skyve Co., LTD. and Skyve R&D Lab. Skyve developed the rehabilitation protocol program, Sagarvision VR, used in this study.

